# The biopsychosocial model: not dead, but in need of revival

**DOI:** 10.1192/bjb.2022.29

**Published:** 2022-08

**Authors:** Simon Williamson

**Affiliations:** 1 University of Warwick, UK; 2 Coventry and Warwickshire Partnership NHS Trust, UK

**Keywords:** Biopsychosocial model, formulation, clinical practice, core values, holistic care

The biopsychosocial model, formalised by Engel in 1977, is at its core an acknowledgement that biological, psychological and social factors causally influence health and disease.^1^ The word ‘model’ is broadly defined by Engel as ‘nothing more than a belief system utilized to explain natural phenomena, to make sense out of what is puzzling or disturbing’. In this sense, ‘paradigm’ may be a more appropriate term.^2^ Indeed, a paradigm shift in psychiatry has occurred since Engel's original paper, with a biopsychosocial framing now cemented in education, training and the Royal College of Psychiatrists’ core values.^3^ Despite its widespread adoption, the model is far from uncontroversial. Criticisms are multi-levelled, from philosophical underpinnings through to application in clinical practice. Below is an assessment of the fundamental challenges the biopsychosocial model faces. Although the model is not dead in any paradigm-shifting sense, significant challenges remain in translating it to practice effectively, requiring more than mere statements of value.

In his original paper, Engel argued that the biomedical model of the day had become a ‘cultural imperative’; a framework so embedded in medicine that its limitations were not easily discernible. Likewise, the embedding of the biopsychosocial model within core values suggests that it too has now reached a similar status in Psychiatry. Therefore, in the interests of being undogmatic, the model will be discussed through the lens of three fundamental questions: is it valid, is it useful and is it used?

## Philosophical coherence (is it valid?)

The primary philosophical challenge for the model is an ontological one. Engel, in expanding the biomedical model, proposed that psychological and social events actually cause illness, and are not merely irrelevant epiphenomena.^1^ As a result, he was burdened with explaining the nature of causation at the psychological and social levels, where reductionist explanations in terms of molecular processes are simply not tenable. To achieve this, Engel appealed to general systems theory, which challenged reductionism by theorising the existence of emergent behaviour, and therefore emergent causal mechanisms.

In their recent book, Bolton and Gillett expand on Engel's systems approach in light of modern scientific developments.^4^ They illustrate how biological cells operate using regulatory frameworks that, despite absolute obedience to the laws of physics and chemistry, are capable of manipulating physico-chemical events for their own continued survival. This occurs in the same manner, they state, as a factory that utilises conveyers, gates and specific raw materials to ensure a smooth manufacturing process. Crucially, causation can be attributed to the regulation itself, and not simply the physical laws being regulated. Since biological, psychological and social phenomena can all be expressed in terms of regulatory systems, there is cohesion of causality between them.

The regulatory framework itself is also worth considering. As we move up the hierarchy from micro to macro, each new level allows for new forms of organisation and diversity in regulating the levels beneath it, i.e. new causal processes. In this manner, biological systems arise to regulate the behaviour of physico-chemical processes, psychological systems arise to regulate the behaviour of biological systems and social systems arise to regulate the behaviour of many individual psychological systems (i.e. people). Although higher levels regulate lower ones, each new level's proper functioning is entirely dependent on the lower levels from which it has risen. With such a framework, interactions between biopsychosocial factors can be theorised coherently, as existing in the same ontological space. Bolton and Gillett note that the framework is also wholly complementary to modern proposals of an embodied brain; with the brain considered as arising from a pre-existent biological system.

## Theoretical utility (is it useful?)

Before addressing questions of implementation, it is worth considering whether the conceptual framework outlined above is of use in principle. Ghaemi would propose not.^5^ He charges Engel's expansion of the biomedical model with adherence to a ‘more is better’ philosophy, where reductionism is wrongly abandoned in favour of searches for complex, multi-levelled mechanisms (i.e. additive eclecticism). Peptic ulcer disease, Ghaemi recalls, was long considered a psychosomatic illness before the discovery of *Helicobacter pylori*. The biopsychosocial model, however, need not abandon reductionism as a scientific method. To describe something as more than the sum of its parts is not to deny the existence of the parts. The model simply speaks to the broader nature of potential causes: not ‘more is better’, but simply ‘more is there’.

As Ghaemi's example demonstrates, reductionist approaches are often the most viable method of initial investigation (although within the framework above they may be better described as lower-level approaches, to avoid confusion with the ontological position of reductionism). Engel's original sentiment, echoed by Bolton and Gillett, is in fact consistent with this.^1^ However, they propose that although reductionistic approaches worked spectacularly well for the biomedical model in its time, today's most prevalent diseases are increasingly aetiologically complex, and therefore less suited to such an approach. The ‘low-hanging fruit’ of infectious diseases (such as *H. pylori*) suited the biomedical model; mental illness does not.

A second charge from Ghaemi, among others,^4^ is that the model is simply too vague to be useful. As Ghaemi puts it, using a biopsychosocial framework to individualise patient treatment ‘has come to mean, in practice, being allowed to do whatever one wants to do’. In other words, since the model specifies no method for its implementation, clinicians will default to whichever aspect of the biopsychosocial they prioritise, resulting in the simple reinforcement of existing dogmas. Or, as Ghaemi puts it, ‘eclecticism produces dogmatism’.

To say that the model can result in dogmatism, however, is not to say that it must. Formulation, for instance, with the addition of the popular 4 Ps framework,^6^ is well-known as a tool that encourages clinicians to comprehensively consider all domains of the biopsychosocial. To engage in this process and continue to maintain a unidimensional focus would be dogmatism in spite of the model, not because of it. Indeed, use of formulation can improve clinician understanding of a patient and, in turn, their management plan, as is aptly demonstrated by Klein's early investigation of panic disorder.^7^ That both negative outcomes (such as Ghaemi's) and positive ones (such as Klein's) can arise from applying the model suggest that the challenge remains not in its theoretical utility, but in its actual use. The biopsychosocial model, therefore, lives or dies on its implementation.

## Practical relevance (is it used?)

Formulation is still the primary tool for clinicians to consider individuals using a biopsychosocial framework. To what extent, then, is it used in practice? Mohtashemi et al's study on the perceptions of psychiatrists is enlightening.^8^ Many of those interviewed, with varying levels of experience, held the view that formulation was distinctly secondary to diagnosis and medication in practice: ‘if someone is bipolar, it's bipolar, you know they're manic, you don't need to [formulate] … you do [a] diagnosis’. It was also reported that, when pressured for time, formulations were often not completed at all. The picture put forward is one of formulation as an afterthought; unlikely to be completed regularly, and far from a core component of practice.

The view quoted above suggests that the biopsychosocial model is practically irrelevant, or rather that its relevance is not appreciated. To propose that formulations are unnecessary, even in the care of acute mania, is to deny the patient their individuality. Formulation is a prerequisite to understanding the individual and their unique circumstances. Understanding in turn improves the therapeutic relationship, leading to better and more collaborative outcomes.

The view of psychotropic management as more important than psychosocial management is also apparent when considering the systems in place for their delivery. Medication is an imperative: prescription charts are signed, and administration will follow. No such sign off is required for psychological or social interventions, and their likelihood of occurrence once suggested is lower. Quite simply, the provision of truly biopsychosocial care in practice is lacking.

## Conclusions

There is a disconnect between the education we receive, the competencies we are trained in and the psychiatry that we practice. The biopsychosocial model is valid and useful, but it can be of no use to our patients if we fail to implement it. As such, a concerted effort is required to revive it in practice. Senior psychiatrists should lead by example and formulate, with the appreciation that individuals are more than their diagnoses. Moreover, this should be done regularly to reflect the ever-changing nature of biopsychosocial dynamics.^9^ Trainees should also formulate, and embrace the advancement of psychiatry toward more holistic care. Crucially, formulations need to be documented if we are to move the model from the realm of handwaving to that of concrete plans. The biopsychosocial model will always be of significance to the history of psychiatry. It is therefore encouraging to note that, within the recently updated curricula of the Royal College of Psychiatrists,^10^ it remains of core value. Let us strive to truly value it.

## About the author

**Simon Williamson** (MBBS, MPhil) is an Academic Clinical Fellow in General Psychiatry at the University of Warwick, UK, and a Core Psychiatry Trainee with Coventry and Warwickshire Partnership NHS Trust, UK.
